# A data-driven performance dashboard for surgical dissection

**DOI:** 10.1038/s41598-021-94487-9

**Published:** 2021-07-22

**Authors:** Amir Baghdadi, Sanju Lama, Rahul Singh, Hamidreza Hoshyarmanesh, Mohammadsaleh Razmi, Garnette R. Sutherland

**Affiliations:** 1 Project neuroArm, Department of Clinical Neurosciences, and Hotchkiss Brain Institute, University of Calgary, Calgary, AB Canada; 2 Binder Dijker Otte (BDO) Canada LLP, Calgary, AB Canada

**Keywords:** Computational biology and bioinformatics, Neuroscience, Health care, Medical research, Engineering

## Abstract

Surgical error and resulting complication have significant patient and economic consequences. Inappropriate exertion of tool-tissue force is a common variable for such error, that can be objectively monitored by sensorized tools. The rich digital output establishes a powerful skill assessment and sharing platform for surgical performance and training. Here we present SmartForceps data app incorporating an *Expert Room* environment for tracking and analysing the objective performance and surgical finesse through multiple interfaces specific for surgeons and data scientists. The app is enriched by incoming geospatial information, data distribution for engineered features, performance dashboard compared to expert surgeon, and interactive skill prediction and task recognition tools to develop artificial intelligence models. The study launches the concept of democratizing surgical data through a connectivity interface between surgeons with a broad and deep capability of geographic reach through mobile devices with highly interactive infographics and tools for performance monitoring, comparison, and improvement.

## Introduction

According to the World Health Organization (WHO), surgical procedures lead to complications in 25% of patients (around 7 million annually) among which 1 million die^1^. Among surgical tasks responsible for error, tool-tissue force exertion is a common variable. Studies on simulation and live surgery have shown a meaningful relationship between surgical errors and inappropriate use of force contributing to an annual cost of over $17 billion in the USA alone which may be avoided by proper monitoring and feedback mechanisms^2–4^. Using SmartForceps System, we have shown that high force error is associated with bleeding, low force error with the need to repeat the task, and force variability, with both bleeding and the need to repeat the task^5^. To enhance surgical safety early on, i.e. training phase of surgeons, established methods of surgical evaluation ranges from mandated Accreditation Council for Graduate Medical Education (ACGME) end of surgical rotation written feedback to intra-operative discussions and guidance^6^. The former has limitations in that the assessment feedback to trainees occur after the rotation and may suffer from observer bias. The latter, on the other hand, can be distracting for surgical trainees especially during the early phases of their learning curve as they are distressed with high intra-operative cognitive tensions.

Subjective surgical skill evaluation including app-based models to rate and provide surgical feedback have been developed, however, these are off-line methods with no attention to task specificity, dependent on expert surgeon supervision for a high-quality assessment, and prone to bias among evaluators^7–9^. Quantitative methods through mobile/web applications offer objective evaluation of surgical skill. Sensor-based systems focusing on hand gesture monitoring have been developed with the goal of capturing technical finesse in surgery^10–12^. The studied sensorized bipolar forceps and the respective data models, allow for real-time monitoring of tool-tissue interaction force profiles, threshold-based high-force recognition, and the ability to discriminate surgeons by their skill level^5^. The prototype SmartForceps System lacked artificial intelligence capabilities to perform the respective force profile monitoring for pattern recognition and surgical skill evaluation.

Artificial intelligence (AI) is remodelling every industry at a rapid pace, and in healthcare, where digital data is becoming increasingly commonplace, its impact can be transformative. The human-oriented revitalization of healthcare AI ranges from improving the quality of life to early disease diagnostics, optimizing the treatment options, and digitizing surgical procedures. Through medical image processing and data-driven models from digital biomarkers, AI applications have shown promise for rapid diagnostics by improving the workflow and automating interpretation of health data, including, enabling patients to monitor their health status through personal devices^13,14^. For performance assessments and patient monitoring in the operating room (OR), however, the AI-oriented and data-driven technologies are lagging behind their counterparts in other healthcare sectors^15–17^. Traditionally closed-door and inherently stressful with rapid decision-making demands, current systems of performance evaluation are limited to surveys for qualitative assessments, manual rehabilitation status or performance parameter tracking, and simulation-based training paradigms^9,18–22^.

Here, we develop a novel data framework for transforming the status quo in the OR environment into a digitized nature, by leveraging sensor-based technology and bringing the power of data and AI into the hands of surgical team for increasing the safety of surgery. This architecture encompasses novel intraoperative parameter monitoring that leads to a transformational surgical education and standardization of patient care. We have developed a data-driven surgical paradigm whereby pattern recognition in the forces of tool-tissue interaction along with sensor-based characteristics of surgical manoeuvres and task completion index serve as objective assessment metrics for surgical competency. In such a platform, the surgical team can review their detailed performance report and compare to the gold standard in an interactive environment*,* where the force profiles of master surgeons reside. By integrating post-operative performance tracking and intra-operating feedback capabilities, we showed the opportunity in leveraging digital data and AI for automated and objective performance tracking using surgical devices which can ultimately benefit patients by reducing complications and increasing safety.

## Results

### Nature of data and app interface

**Figure 1:**
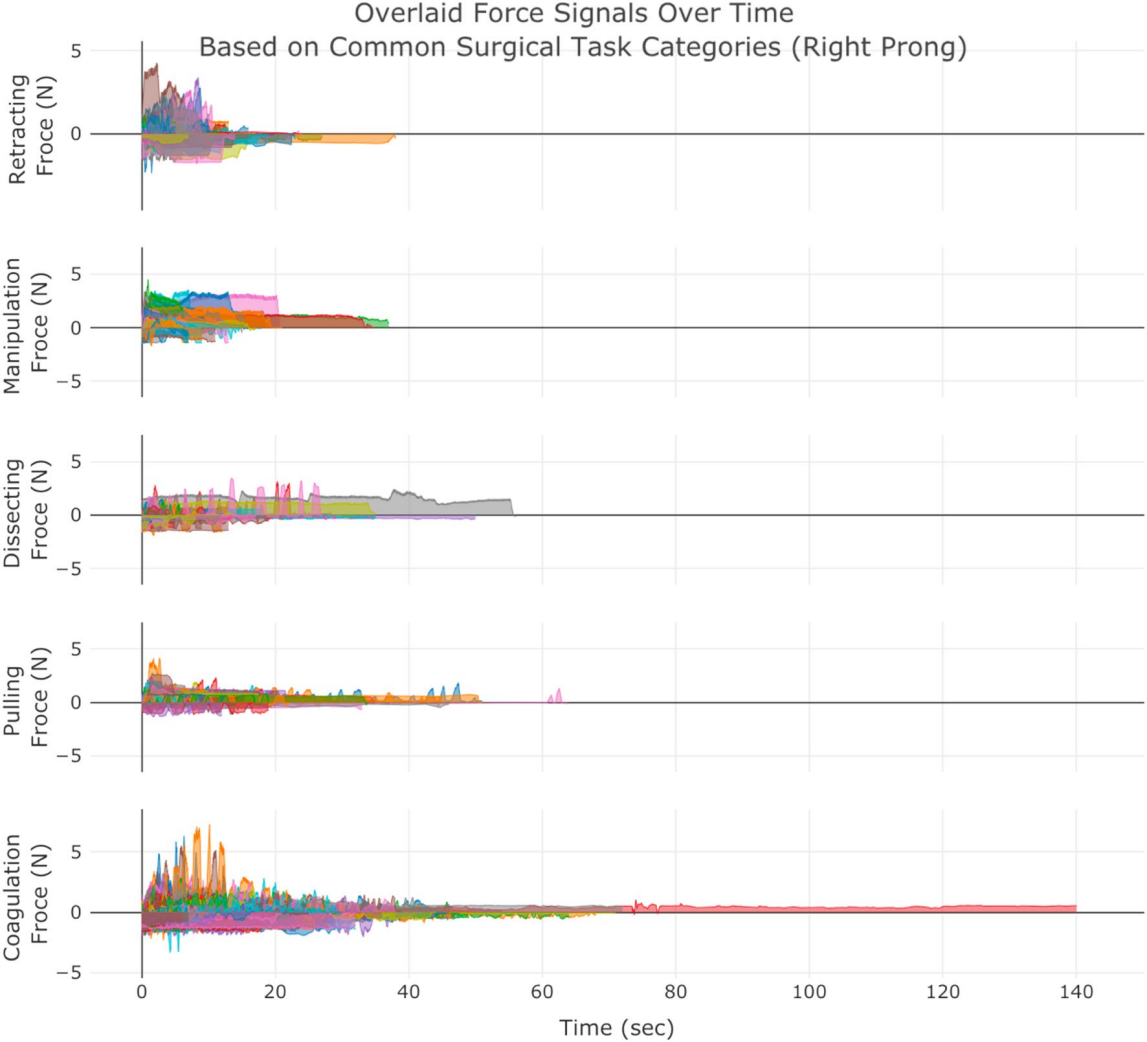
SmartForceps timeseries data of the Right prong across the 5 surgical tasks of Retracting, Manipulation, Dissecting, Pulling, and Coagulation overlaid for 50 cases. Differences in the range and duration of force are shown in the overlaid data profiles. Please refer to our Supplementary Materials for both left and right prong data. These charts have fully interactive capability including zoom, pan, download, etc. Figure created by R Plotly library version 2.0: https://plotly.com/r/.

**Figure 2:**
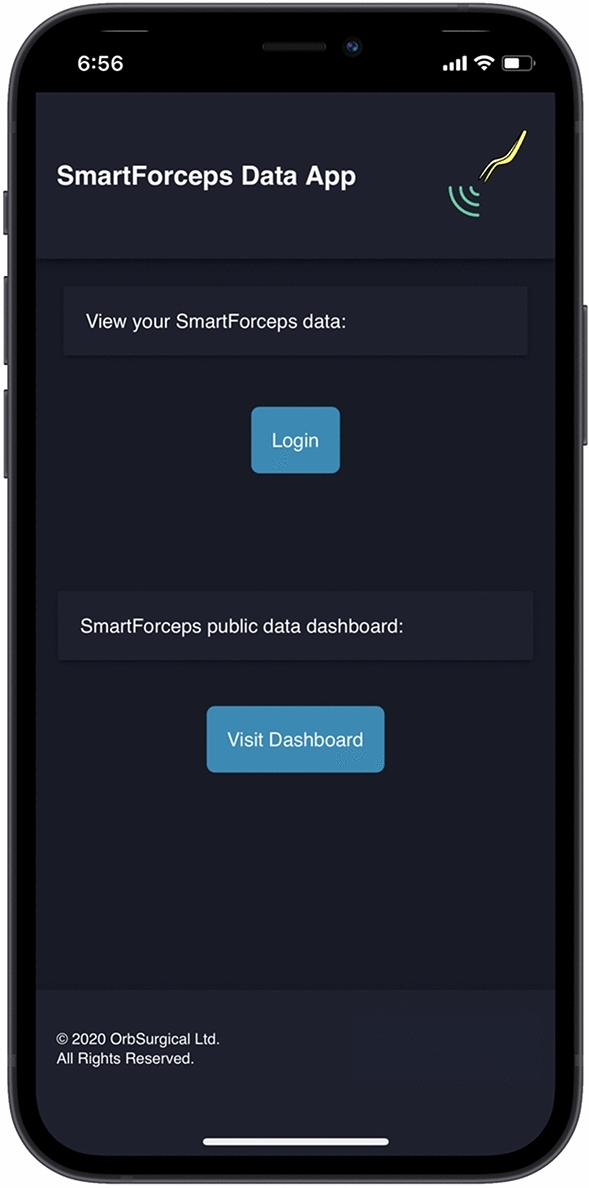
Design interface of the SmartForceps surgical data monitoring application on a mobile device. The user can click to visit the general dashboard without login or login with exclusive credentials to visit their own reports in surgeon or data scientist views. Visualization created through framing the PWA created in JavaScript inside a phone view using MockuPhone mock-up generator: https://mockuphone.com.

**Figure 3:**
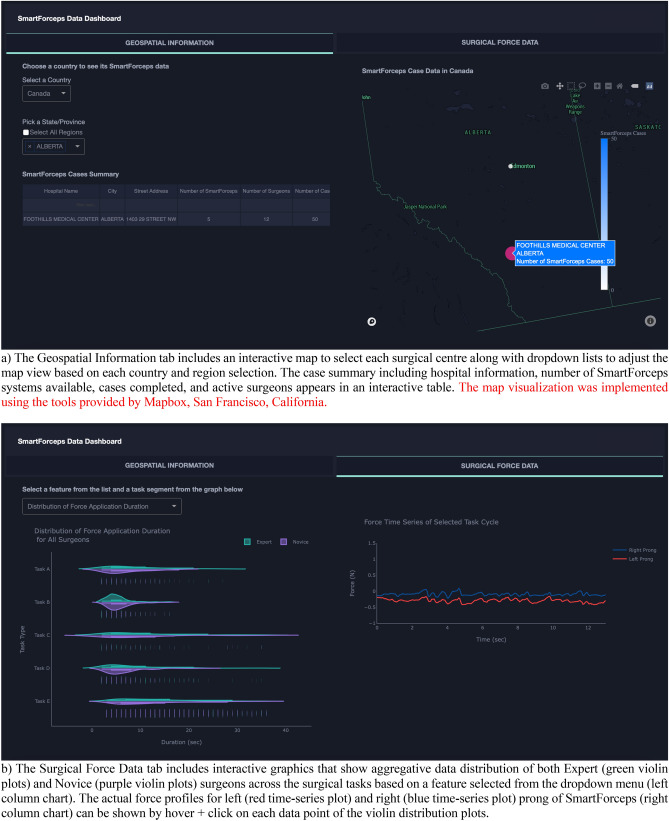
SmartForceps Data Analytics Dashboard in "General" view shown in a desktop mode. The current view includes two tabs of "Geospatial Information" (**a**) and “Surgical Force Data” (**b**). These charts have fully interactive capability including zoom, pan, download, etc. The figure for dashboard was created by Python Dash library version 0.43.0: https://plotly.com/dash/.

**Figure 4:**
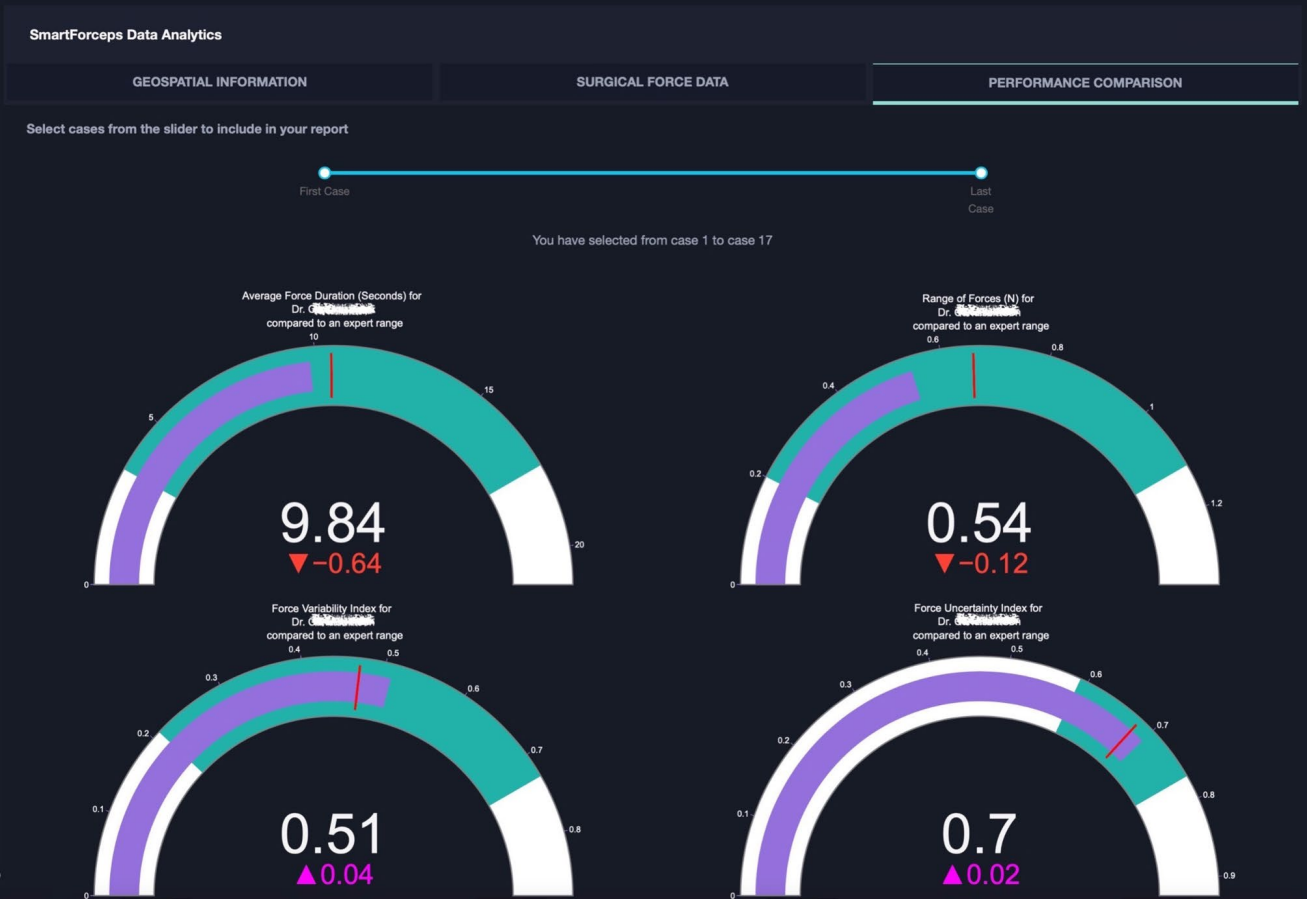
SmartForceps Data Analytics Dashboard in "Surgeon" view. The current view includes three tabs of "Geospatial Information", “Surgical Force Data”, and “Performance Comparison Dashboard”. These charts have fully interactive capability including zoom, pan, download, etc. This figure shows the overtime performance report (with the slide bar at the top to select range of cases) for a Novice surgeon with PGY > 4. The name is deidentified for privacy reasons. The gauge charts show the performance (purple bar) compared to the Expert surgeon (mean and standard deviation indicated as red mark and green area, respectively). In this graph, the representative surgeon gauge starts from zero as the baseline with the goal of reaching to the expert level values denoted by a red bar and green area. The figure for dashboard was created by Python Dash library version 0.43.0: https://plotly.com/dash/.

**Figure 5:**
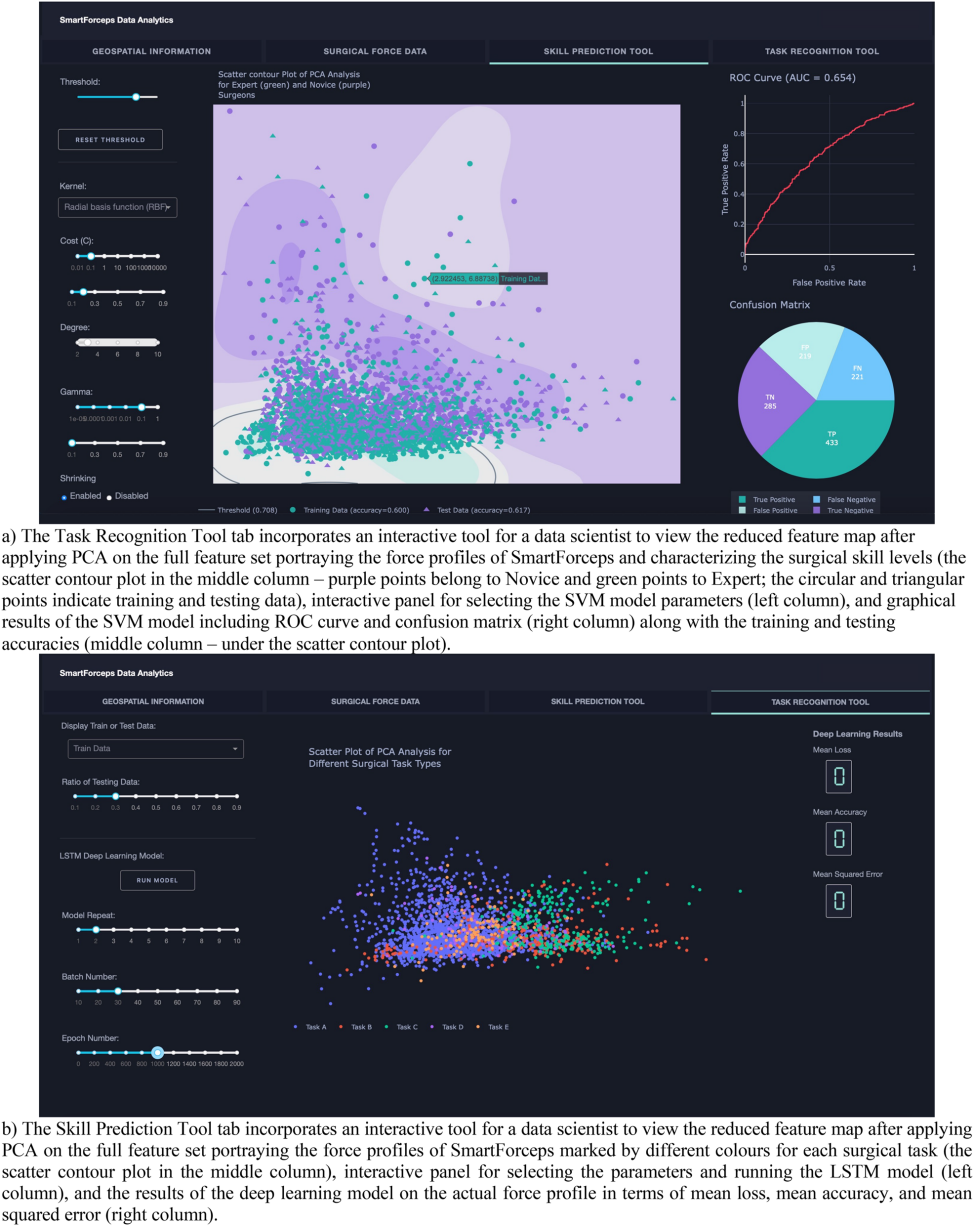
SmartForceps Data Analytics Dashboard in "Data Scientist" view. The current view includes four tabs of "Geospatial Information", “Surgical Force Data”, “Skill Prediction Tool” (**a**), and “Task Recognition Tool” (**b**). The figure for dashboard was created by Python Dash library version 0.43.0: https://plotly.com/dash/.

A snapshot of aggregated force data over the 50 cases of neurosurgery is provided in Fig. 1. The graph can highlight the differences in completion time and range of forces across the 5 surgical tasks. A web/mobile application was developed to monitor the force related data/features and the mobile interface is shown in Fig. 2 shows the interface on a mobile device. The dashboard is personalized for data scientists as well as each surgeon’s view who need to login through their personified credentials to perform data analysis or track their performance by comparing to expert surgeon(s) in the “Expert Room” (Figs. 3, 4, 5). The application contains multiple tabs including (1) For both data scientist and surgeon: Geospatial Information for SmartForceps cases across the world with multiple choice selection lists and interactive maps to display the information in a searchable table (Fig. 3a); (2) For both data scientist and surgeon: Surgical Force Data for visualizing different engineered features across each task through interactive distribution plots showing detailed statistics for Expert or Novice surgeons to compare and reproduce each force segment through mouse hover and click (Fig. 3b); (3) For surgeon: Performance Comparison Dashboard for tracking of individual performance over time characterized by task completion time, range of force application, force variability index, and force uncertainty index (level of entropy in time series data) compared to the average and range of an expert surgeon (Fig. 4); (4) For data scientist: Skill Prediction Tool that provides the necessary elements and gadgets for a step-by-step training and testing of support vector machine (SVM) models with parameter fine-tuning and generating results to distinguish surgical expertise (Fig. 5a); and (5) For data scientist: Task Recognition Tool for visualizing, training and testing of long short-term memory (LSTM) deep learning model with parameter fine-tuning and generating results to perform surgical task classification (Fig. 5b).

### Statistical data analytics

Data were analysed prior to dashboard visualization and data modelling experimentation for a better behaviour understanding of the force profiles. Summary statistics were extracted for each task and surgeon experience that included the number of force segments and mean (SD) of the force features across all available segments. The features used for statistical analysis are provided in Table 1. These hand-crafted features following inspections through statistical analysis will be used in-part or as an augmentation to the future machine learning models.

**Table 1:** Various features were extracted from the force signal data of each prong in SmartForceps to be used in the monitoring models for surgeon skill assessment.

Derived force signal features	Description
Force duration	Duration of force application in one task segment
Force average	Average of force values for one task segment
Force max	Maximum of force values in one task segment
Force min	Minimum of force values in one task segment
Force range	Range of force values across one task segment
Force median	Median of force values for one task segment
Force SD	Standard deviation of force values in one task segment
Force CV	Coefficient of variation of force values in one task segment
Force mean CI (0.95)	Confidence interval on the mean with 95% probability
Force data skewness	The extent to which the force data distribution deviates from a normal distribution
Force data skewness 2SE	The significance of skewness in force data based on dividing by 2 standards errors (significant when > 1)
Force data kurtosis	The extent to which the force data distribution is tailed in a normal distribution
Force data kurtosis 2SE	The significance of kurtosis in force data based on dividing by 2 standards errors (significant when > 1)
Force data normality	Shapiro–Wilk test of normality in force data distribution
Force data significance of normality	Significance of Shapiro–Wilk test of normality
Force peak value	Peak force value in one task segment
Force peak counts	Number of force peaks in one task segment
1st derivative SD	Standard deviation for the first derivative of the force signal in one task segment
Force signal flat spots	Maximum run length for each section of force time-series when divided into ten equal-sized intervals
Force signal frequency	Dominant time-series harmonics extracted from Fast Fourier Transform (FFT) of force value in one task segment
Force cycle length	Average time length of force cycles in one task segment
Force signal trend	Force time-series trend in one task segment
Force signal fluctuations	Force time-series fluctuation index in one task segment
Force signal spikiness	Force time series spikiness index (variance of the leave-one-out variances of the remainder component) in one task segment
Force signal linearity	Force time-series linearity index (from Teräsvirta’s nonlinearity test) in one task segment
Force signal stability	Force time-series stability index (variance of the means) in one task segment
Force signal lumpiness	Force time-series lumpiness index (variance of the variances) in one task segment
Force signal curvature	Force time-series curvature index in one task segment (calculated based on the coefficients of an orthogonal quadratic regression)
Force signal mean shift	Force time-series largest mean shift between two consecutive windows in one task segment
Force signal variance shift	Force time-series largest variance shift between two consecutive windows in one task segment
Force signal divergence	Force time-series divergence index in one task segment (largest shift in Kulback-Leibler divergence between two consecutive windows)
Force signal stationary index	Force time-series stationary index around a deterministic trend in one task segment (based on Kwiatkowski-Phillips-Schmidt-Shin (KPSS) unit root test with linear trend and lag one)
Force signal entropy	Force time-series forcastabilty in one task segment (low values indicate a high signal-to-noise ratio)
First autocorrelation minimum	Time of first minimum of the autocorrelation function in force time-series signal from one task segment
First autocorrelation zero	Time of first zero crossing of the autocorrelation function in force time-series signal from one task segment
Autocorrelation function E1	First autocorrelation coefficient from force time-series signal in one task segment
Autocorrelation function E10	Sum of the first ten squared autocorrelation coefficients from force time-series signal in one task segment

This table provides a full list of these time-series and statistical features along with their detailed definitions.

The number of force segments were 2085 for Coagulation (Expert: 1108; Novice: 977), 303 for Pulling (Expert: 192; Novice: 111), 296 for Manipulation (Expert: 210; Novice: 86), 89 for Dissecting (Expert: 64; Novice: 25), and 122 for Retracting (Expert: 71; Novice: 51), with a total value of 1645 for Expert and 1250 for Novice surgeons. The mean (SD: Standard Deviation) for *Force Duration* in Coagulation was 12.1 (7.2) seconds—around 58% higher than the average of completion time in other tasks—while the completion time in Pulling, Manipulation, Dissecting, and Retracting tasks were 7.6 (5.3), 5.4 (2.5), 10.1 (8.6), and 7.6 (5.1) seconds, respectively. The mean (SD) for *Force Range* in Manipulation was 1.2 (0.5) N—around 52% higher than the average of completion time in other tasks—while the range of forces in Coagulation, Pulling, Dissecting, and Retracting tasks were 0.7 (0.5), 1 (0.6), 0.9 (0.5), and 0.7 (0.4) N, respectively. For presenting the level of force variability, *Standard Deviation* was calculated across the tasks and surgeons. The mean (SD) across all tasks were 0.23 (0.14) for Expert and 0.27 (0.14) for Novice surgeons. For materializing the unsafe force application risk, *Force Peak Values* were identified across the tasks and surgeons. The mean (SD) across all tasks were 0.35 (0.27) for Expert and 0.39 (0.29) for Novice surgeons. Level of *Force Signal Entropy* was used to measure the level of randomness in force application for among different surgical experience. Mean (SD) of this feature for Expert surgeon was 0.67 (0.09) and for Novice surgeons was 0.65 (0.07). The detailed summary statistics across all features are available at our Supplementary Materials.

The ANOVA test results for inspecting the holistic relationship between the generated features and the variables of interest, i.e., skill level and task type, showed significant difference between experience levels in various features including *Force Maximum (p* < *0.001), Force Range (p* = *0.001), Force Standard Deviation (p* < *0.001), Force Distribution Kurtosis (p* = *0.001), Force Peak Values (p* = *0.001), Force Flat Spots (p* < *0.001), Force Signal Frequency (p* = *0.001), Force Signal Fluctuations (p* = *0.02), Force Signal Stability (p* = *0.001), Force Signal Mean Shift (p* < *0.001), and Force Signal Entropy (p* = *0.001).* Among various tasks, several features were significantly different, e.g., *Force Duration (p* < *0.001), Force Average (p* < *0.001), Force Maximum (p* < *0.001), Force Range (p* < *0.001), Force Peak Values (p* < *0.001), Force Peak Counts (p* < *0.001), Force Signal Flat Spots (p* < *0.001), Force Signal Frequency (p* < *0.001), Force Signal Fluctuations (p* < *0.001), and Force Signal Stability (p* < *0.001),* and *Force Signal Curvature (p* < *0.001).* The results showed no significant difference for *Force Coefficient of Variation* and *Force Signal Cycle Length* among tasks, experience levels, and their interaction. The comprehensive tables for ANOVA tests and post-hoc analysis results for each feature are available in the provided Supplementary Materials.

### Skill classification and task recognition tools

Based on the ANOVA test results, a subset of features was extracted for developing machine learning models. In this subset, *Force Duration, Force Minimum, Force Coefficient of Variance, Force Data Skewness, Force Data Skewness 2SE. 1st Derivative SD, Force Peak Counts, Force Cycle Length, Force Signal Spikiness, Force Signal Stationary Index, First Autocorrelation Zero, and Autocorrelation Function E10* were excluded. In addition, the surgical tasks were classified as 5 main categories of Retracting [the tumour or tissues], Manipulation [of cotton], Dissecting [the tumour or tissues], Pulling [the tumour or tissues], and Coagulation [the vessels/veins in tumour or tissues].

The skill prediction tool provides an interactive environment to explore different models and reach an optimal model for the problem. The preliminary experiments based on the available data of 50 cases using SVM model on 25 extracted features after dimensionality reduction by principal component analysis (PCA) showed the highest area under the curve (AUC) of 0.65, training accuracy of 0.60, testing accuracy of 0.62 with the sensitivity of 0.66 and specificity of 0.57. The optimal model parameters were radial basis kernel function with both cost and gamma values of 0.1 × 10^0.1^.

The task recognition tool also provides an intuitive interface to optimize a deep learning model based on LSTM that has the input layer with 100 inputs, a single layer hidden layer with 100 LSTM neurons, a dropout layer with the ratio of 0.5 to reduce overfitting of the model to the training data, a dense fully connected layer with 100 neurons and ReLU activation function to interpret the extracted features by the LSTM hidden layer, and an output layer with Softmax activation to make predictions for the 5 classes. The optimizer used to train the network was the *adam* version of stochastic gradient descent with *categorical cross entropy* as the loss function. As a showcase example, the preliminary network was experimentally trained for 1000 epochs with a batch size of 20 samples that showed mean (SD) loss of 0.598 (0.001), mean (SD) accuracy of 0.828 (0.001), and mean squared error of 0.055 (0.001).

## Discussion

The study introduces the concept of democratizing exclusive and closed OR data into a globally accessible social domain among surgeons through a secure and personalized monitoring application containing highly interactive features and infographics. The results, while preliminary, denote a powerful set of highly engineered features showing significant distinction between the surgical tasks and skill level of surgeons. Based on this significance, through stringent data-driven hypotheses and analytics, AI models for task-specific and real-time performance monitoring system and post-operative skill assessment and improvement were examined. The results were further confirmed with the rate of at least 66% for true expert recognition using a set of 25 engineered time-series features and a mean accuracy of 83% across 4 model repeats for task type recognition using a deep learning model.

The increasing popularity of professional and social connectivity through secure mobile applications has been welcomed by healthcare professionals. These technologies can be used to improve the standard of care and direct personalized education to surgical residents^22^. In surgical education, social media provides a communication tool for daily discussing and intake of targeted information in their social feeds regarding surgical techniques. This practice seems ideal by promoting an economical and efficient method of information sharing and continuous feedback on performance. However, the credibility of such information must be validated by going through scrutiny by professional entities^23^. The idea of *Expert Room* entails all the perks and privileges in ordinary social networks in terms of group discussion and, in addition, introduces an objective and interactive environment for obtaining case-specific feedback and data-driven analytics immediately after each surgical procedure, reviewing the progress, and sharing *the goods* (exciting and valuable information) with other peers in the field.

Visualization of data analytics of complex nature is pivotal for ease of interpretation and better understanding, thus optimizing the educational as well as technical message to surgeons. The human’s high throughput visual perception channel allows a convenient way for connecting data representation to existing or new knowledge through visualization^24^. The significant transformation on the internet and mobile applications has redefined data visualization by enabling complex multi facet infographics with high interactive capabilities, and medical data representation must follow this streamline.

The implications of artificial intelligence on medical data given the abundance of collected data in digitized healthcare systems are promising. However, security concerns and segregation of data for individual hospitals impedes the full-scale utilization of machine learning capabilities by diversifying data from distinct data generation centres, thus posing limitations for transition from research to clinical practice^25^. Through secure and encrypted model implementation schemas, e.g., federated learning, data modelling parameters (instead of the actual data) learned in each medical centre, carrying the characteristics of surgical finesse is expected to pass hospitals firewall and will aggregate in the cloud environment. In this framework, data privacy for both surgeons and patients, has been satisfied by the inclusion of non-critical informed data with no direct patient or surgeon personal information processed. Data privacy is further ensured by performing cloud-based data management and analytics and hosting the web application through Azure in which the security is safeguarded by Microsoft’s established Health Insurance Portability and Accountability Act (HIPAA)-compliant data protocols. In addition, the personalized profiles for performance tracking are user-specific and authenticated through organizational secure accounts as a top-layer security before revealing or accessing any related OR information. The trained artificial intelligence model could be easily shared across healthcare centres, as pure model parameters, bypassing the privacy concerns of sharing sensitive personal data.

Leveraging artificial intelligence in medicine has no intention to disregard human capability, but rather as an enabler that strengthens the ability and allows more time for specific elements of their job that require emotional intelligence^26^. The utilization of intelligent framework for proficiency evaluation would enable all surgeons to access an equivalent level of assessment as if the best panel of surgeons would perform such assessment^27^. Through democratization or sharing of surgical data in the form of Expert Room/Master Surgeons environment and proper implementation techniques, e.g., federated learning, here we present a pathway towards a smooth transition to clinics which can be welcomed by both hospitals and medical communities. Such a solution, through localized and secure treatment of data, addresses privacy and legal challenges. In the cloud modelling aggregation environment, machine learning parameters from various centres containing the specifics belonging to different geolocations with various medical training standards will be combined to avoid bias from unbalanced data. In such a model, a surgeon from remote parts of the world is granted the opportunity to objectively compare one’s quantified performance with counterparts in advanced centres without having any interaction or knowing the panel of expert surgeons whose data are aggregated in this environment. We believe that such a platform can democratize operating room data, surgical experience, and medical education.

The present study is unique as it emphasizes the opportunity in harnessing the data derived from quantifying the technical performance of surgery and sharing it securely and immediately with those outside the OR, landing novice surgeons to a non-intimidating virtual and safe environment. By collecting, storing, and centrally retrieving surgical data stream, this report demonstrates the power of data and AI through returning performance metrics back to surgeons’ fingertips, thus immortalizing surgical skills, making it both transferable and accessible, without geographical or historical restrictions. Technologies like SmartForceps can empower both surgeons and trainees around the world to learn, adopt and gain from the OR data, review the practical experience of their valued peers, and foster collaboration and gain new insights from the data. Machine intelligence and continuous learning provide an ability to look back into the technical history of surgery, which can improve the collaboration and shared knowledge between surgeons and residents, improve the learning curve for new residents and enable expert surgeons to self-navigate through unique and complex patient cases. Indeed, the ideal combination of real OR experience merged with virtual cloud-based dataroom, enhances both performance and learning of complex and intimidating skills in a seemingly non-intimidating platform. The sensor-driven technology allowing a digital, quantifiable output, is both timely and necessary as the apprenticeship-based surgical training model transitions to a competency-based paradigm.

The work, first of its kind and unique, does face some limitations in the numbers of data points for different task types and currently only one expert surgeon for establishing the comparisons. Our focus in this paper was to provide a showcase to the opportunity of using quantified operating room data for objectively tracking and analysing surgery. While the present study fulfils the examination objective in establishing data management, analytics and artificial intelligence models, the prospective work will continue with an ever-increasing data and metrics across multiple centres, surgeons of various experience, and an expanding population of surgical trainees and procedures which will lead to a comprehensive answer to the problem at-hand through AI-enabled data pipeline and modelling. As time passes, the continual inflow of rich and homogenous dataset would further improve the machine learning model with an outflow of comprehensive analytics and results through ongoing data model design and hyperparameter optimization.

## Methods

### Surgical data collection

Force data of tool-tissue interaction was recorded while performing neurosurgical manoeuvres categorized into 5 main different tasks, i.e. (1) coagulation (cessation of blood loss from a damaged vessel), (2) dissection (cutting or separation of tissues), (3) pulling (moving and retaining tissues in one direction), (4) Retracting (grasping and retaining tissue for surgical exposure), and (5) manipulating (moving cotton or other non-tissue objects), identified through cumulative patient case monitoring and upon expert approval. The study with clinical use of the SmartForceps System was approved by the Conjoint Health Research and Ethics Board of the University of Calgary (REB19-0114), with permission from Surgical Services—Foothills Medical Centre, Calgary, AB, Canada. All methods were performed in accordance with guidelines and regulations per the research protocol outlined and approved within REB19-0114. This approval also included a waiver of written consent. Details on the technology development, pre-clinical and clinical use are available in our previous publications^5,11,28,29^.

The tasks were recorded along with vocals from the operating surgeon annotating the period of force application and specific task name. The voice recordings were used to segment the force time series data and put labels on each segment, facilitating the training phase of machine learning. The recorded time stamps for voice and force data were synchronized, and together with a distinct beep sound being played while the forceps was switched on, facilitated the localization of start and end points for each task segment. Surgical microscope videos served as post-hoc validation for data labelling when necessary.

SmartForceps was employed in 50 neurosurgery cases majority of which were for tumour resection of various types in adult patients including meningioma, glioma, hemangioblastoma, and schwannoma. Twelve surgeons performed the cases, which included one Expert surgeon with 30+ years of experience and 12 Novice surgeons including residents with post-graduate years (PGY) ranging across 3 levels of 1–2 (n = 4), 3–4 (n = 3) and > 4 years (n = 4), and one fellow. The surgical team adopted and used the SmartForceps System, similar to and instead of, a conventional bipolar forceps. The added advantage was the provision of real-time tool-tissue force measurement, display and recording. Details of patient demographics, pathology, location, tumour size and surgeon experience level are provided in Table 2, with detailed information for each case provided in Supplementary Materials.

**Table 2:** Patient demographics, pathology, location, tumour size and surgeon experience level.

Mean (SD) age	54.7 (14.1)
Gender (male/female)	30 M/20 F
Disease type (count)	Hemangioblastoma (3)
	Glioma (10)
	Vestibular schwannoma (15)
	Meningioma (10)
	Cavernous angioma (2)
	Trigeminal neuralgia/hemifacial spasm (4)
	Chiari malformation (2)
	Cervical spondylosis (2)
	Arteriovenous malformation (1)
	Paraganglioma (1)
Location	Frontal (10), temporal (4), parietal (4), occipital (1), brainstem (2), posterior fossa (27), cervical spine (2)
Mean (SD) tumour size (cm, max. diameter R1 × R2 × R3)	3.3 (1.8) × 2.8 (1.5) × 2.8 (1.5)
Surgeon experience year (count)	30 + (1), fellow (1), PGY 5–6 (4), PGY 3–4 (3), PGY 1–2 (4)

### Data processing and statistical analytics framework

The data management framework for SmartForceps incorporates a cloud-based pipeline for acquiring and processing the intraoperative data. Upon securely uploading the case data into our customized Azure data warehouse authenticated through organizational Microsoft credentials, the recorded raw data was orchestrated and transformed through Azure Data Factory pipelines and stored into Blob Storages. The stored data was leveraged in the cloud by Azure Databricks data engineering tools and through R and Python coding languages.

Our data management framework based on custom-built curing pipeline and reporting structure incorporated a data ingestion point where the segmented force profiles representing a consolidated period of force application in a specific surgical task was imported. The force segments were identified through the processing of OR voice data and were concatenated into a structured dataframe containing various information including timestamp, surgeon and experience level, surgical task type, and high/low force error or bleeding instances. Segmented force profile data were pre-processed for noise and outlier removal and to find accurate force peaks within each task segment. The signals were smoothed by passing through a digital 4th order Butterworth low-pass filter with a cut-off frequency of 0.1 Hz. Further, the outlier segmented data were identified based on 1st and 99th percentiles of either maximum force, minimum force, or task completion time from all trials of the expert surgeon as < 1% error was assumed to occur by experienced surgeons. The force segments for which the maximum force peak, minimum force valley, or task completion time exceeded the upper threshold (99th percentile) or fell short of the lower threshold (1st percentile) were labelled as outliers and removed (~ 11%). Thus, the clean, engineered features served as a baseline for hand-crafted feature-based surgical skill stratification.

**Figure 6:**
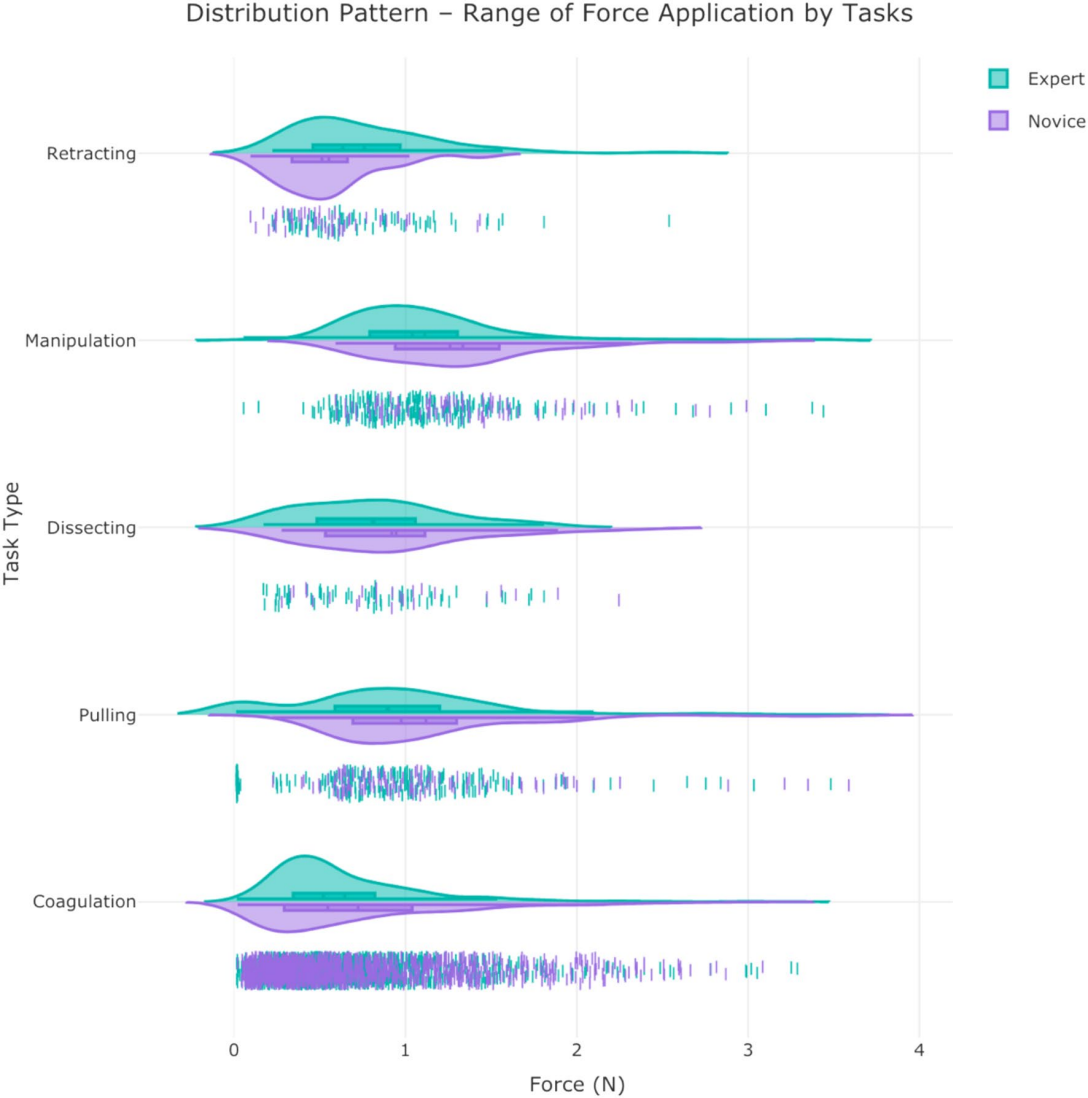
Aggregative data distribution of both Expert (green violin plots) and Novice (purple violin plots) surgeons across the surgical tasks for each time-series extracted feature (Force Range in this sample graph). Detailed statistical information including min, max, median, mean, q1, and q3 are available to view on mouse hover in the original app and the Supplementary Material. Figure created by R Plotly library version 2.0: https://plotly.com/r/.

**Figure 7:**
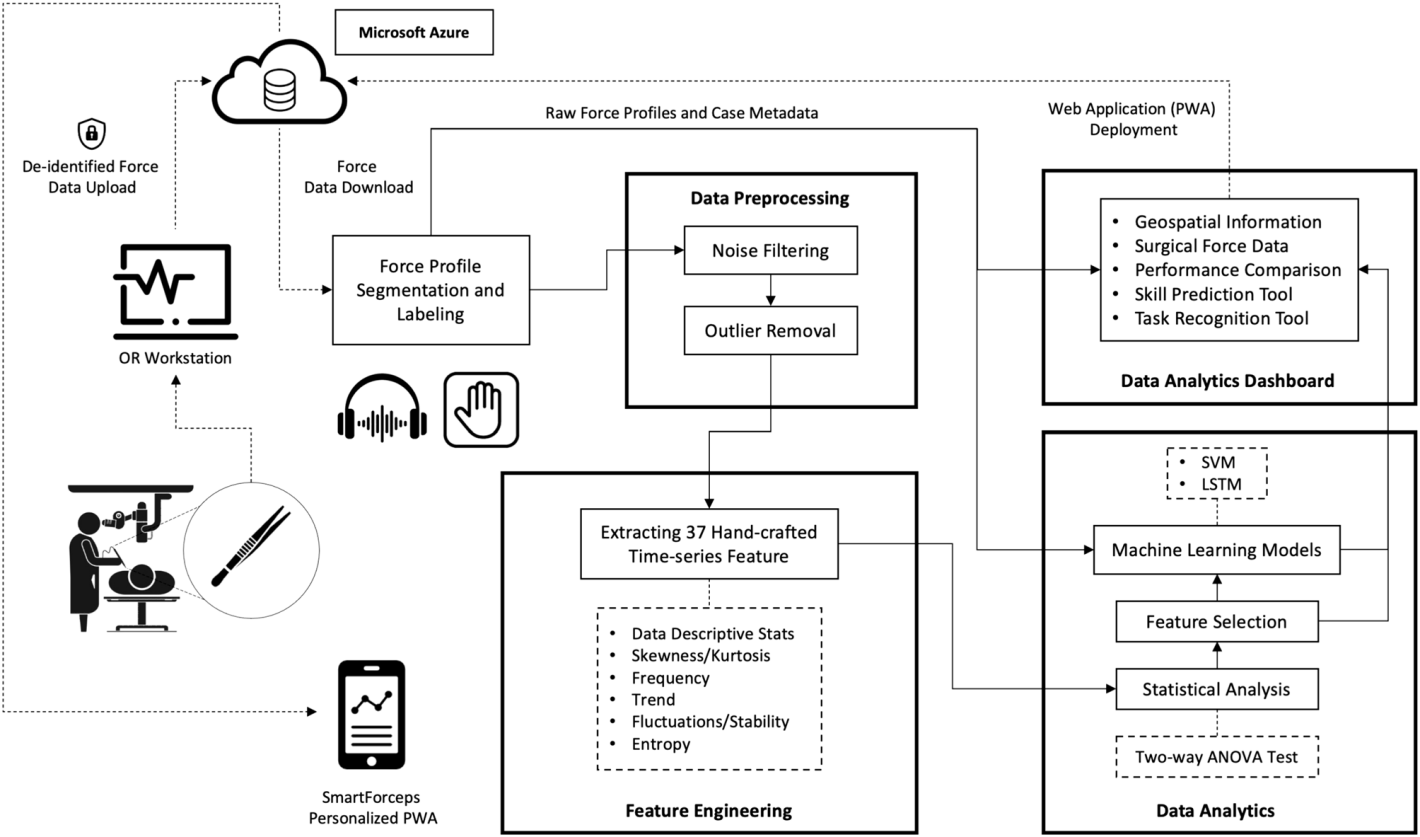
Workflow diagram of SmartForceps data management and analysis pipeline. Forces of tool-tissue interaction along with de-identified case information is uploaded to a HIPAA-compliant data storage and analytics architecture, i.e., Microsoft Azure. Force data were manually segmented and labelled by listening to the operating room voice recordings where each surgeon name, surgical tasks, and important incidents were properly narrated. Data Pre-processing was performed for noise filtering (Butterworth low-pass filter) and outlier removal (1st and 99th percentiles of either maximum force, minimum force, or task completion time). To generate a holistic information from tool-tissue interaction force profiles, 37 hand-crafted time-series features were extracted in Feature Engineering phase. In Data Analytics phase, two-way ANOVA tests were examined to monitor the representation power of each feature set for different surgeon skill and task categories and a subset of 25 features was selected. The force profiles and selected features were used in Data Analytics Dashboard for performance monitoring and machine learning modelling tools to perform skill prediction and task recognition. The visualization was created in Microsoft PowerPoint version 16.49 with the icons obtained from a Google search: e.g., https://www.iconfinder.com.

Statistical tests were designed to understand the pattern of force data in various conditions under investigation. To this end, independent measures two-way ANOVA was performed that simultaneously evaluates the effect of experience and task type as two different grouping variables on the continuous variable of tool-tissue interaction force. A subset of 25 time-series-related variables was selected for the subsequent analysis among the 37 hand-crafted features based on statistical tests to monitor their representation power in different surgeon skill and task categories (Fig. 6). The aim was to have the best explanation of patterns and behaviours for force profiles over the timespan of each data segment (details are available at the Supplementary Materials). Among these features, Force Duration, Force Range, Force Signal Fluctuations (Variability Index), and Force Signal Entropy (Uncertainty Index) were selected as representative measures in the performance dashboard (Fig. 4). The time-series data of force profiles along with the selected hand-crafted features were used for the future development of machine learning models to discriminate surgical skill and recognize performed surgical tasks. Workflow diagram of SmartForceps data management and analysis pipeline is provided in Fig. 7.

### Online data analytics dashboard

The calculated features alongside with the case metadata were passed into a custom-built Dash-Plotly (Plotly, Montreal, Canada) architecture developed in Python environment to construct an interactive web application for visualization and interpretation of data. This platform was interfaced with a progressive web application (PWA) to make it installable on mobile devices. Unlike traditional native mobile applications, PWA incorporates the best of both web and native application worlds in terms of capabilities and the power of reach to anyone, anywhere, and on any device. Dash framework is designed based on Flask and Plotly.js making the application ideal for seamless connection to a Python-based data processing and machine learning platform, enabling a virtually unlimited user interface customization, thus encompassing all the technologies and protocols required for building an interactive web application. Dash is a lightweight and intuitive application that can scale to high levels of traffic, and we have implemented this platform for our public domain to present and interpret SmartForceps data. Detailed personalized data is made available to each surgeon through their user-specific account. In this way, each user will be able to login and view/share their performance and compare their case data with other colleagues in the field. The web application is hosted online through our Azure Service Plan.

## Supplementary Information

Supplementary Information.

## Data Availability

An HTML file based on our R Markdown document has been created and hosted at: https://smartforceps.github.io/supplementary/ that provides a complementary and detailed information categorically presented over the course of technology development. The original data and codes allow investigators to replicate and test this work.
