# Supplementary material for: A data-driven performance dashboard for surgical dissection
Source: Sci Rep. 2021 Jul 22;11:15013. doi: 10.1038/s41598-021-94487-9 (PMC8298519; doi:10.1038/s41598-021-94487-9)
Supplement: Supplementary file 1 — Supplementary Information. [file 41598_2021_94487_MOESM1_ESM.pdf]

Data Recording
SmartForceps Software
Software Demo
Orbsurgical SmartForceps Web Application
Data Processing
SmartForceps Dashboard App

SmartForceps Data Framework

Code

Supplementary Document

Authors blinded for peer review.

June 21, 2021

Force data from Expert and Novice surgeons; data extracted from 50 procedures (multiple tasks per procedure) and segmented for each microsurgical task.

This document contains the data management and analytic framework for SmartForceps, a sensorized surgical bipolar forceps capable of quantifying the forces of tool-tissue interaction in microsurgery. We have shown that high force error is associated with bleeding, low force error with the need to repeat the task and force variability with both. We have further established that novice and intermediate level surgeons exert more force errors compared to experienced. The technology introduces a data-driven surgical paradigm whereby forces of tool-tissue interaction are used as an objective assessment metric for surgical competency. Together with the error warning system, the reduction of surgical error and improved patient safety is promised.

The present document incorporates 50 cases of neurosurgery performed at Foothills Medical Centre, Calgary. The data were extracted and segmented for each task of microsurgery performed by an Expert and multiple Novice surgeons. The details of each case is provided in the table below:

Code

Details for 50 cases of neurosurgery performed using SmartForceps

Case.number	Location	Tumour.Pathology	Tumour.Size_cm	PGY	Age.Sex	Procedure	NOTES
1	Left CP Angle	Vest. Schwannoma	2.1x1.9x2.1	5	46/M	Left Translab exposure for excision of tumour (CP Angle)	
2	Left Cerebellum	Hemangioblastoma	1.5x1.0x1.1	3, 2	39/M	Redo suboccipital craniotomy for excision tumour	VHL Syndrome
3	Left CP Angle	Vestibular Schwannoma	1.6x2.3x1.5	5	43/M	Left Translab exposure for excision of tumour (CP Angle)	
4	Left Temporal Lobe	Glioblastoma - WHO Grade IV	6.2x5.3x5.3	5	67/M	Left Frontotemporal Craniotomy for excision tumour	
5	Left Fronto-temporal	Glioblastoma - WHO Grade IV	5.2x4.5x5.5	5	78/M	Left fronto-temporal Craniotomy for Excision Tumour	
6	Right Frontal Lobe	Glioblastoma - WHO Grade IV	6.2x3.2x4.0	5	56/M	Right Frontal Craniotomy for excision tumour	
7	Right Posterior Parafalx	Meningioma - WHO Grade I	6.1x5.6x5.6	5, 2	66/M	Right Parietal Craniotomy for excision tumour	
8	Midline Posterior Fossa	NA	NA	3, 1	52/M	Suboccipital Craniotomy & removal Posterior Neural Arch C1 for Decompression Chiari Malformation	
9	Right Posterior temporal	Glioma-Astrocytoma WHO Grade III	0.6x0.6x0.6	1	40/F	Redo Right Temporal Craniotomy for Excision of Tumour	
10	Left Trigeminal Neuralgia	NA	NA	3, Fellow	37/F	Redo Left Suboccipital Craniectomy for Microvascular Decompression (CN V-VII-IX-XI)-Glossopharyngeal Neuralgia	
11	Right Trigeminal Neuralgia	NA	NA	5, 2	56/F	Left Suboccipital Craniectomy for Microvascular Decompression	
12	Cervical Spine	NA	NA	5, 1	55/M	C4-5/C5-6 Anterior Cervical Discectomy & Fusion	
13	Right CP Angle	Vestibular Schwannoma	3.1x2.0x2.8	5	63/M	Right Suboccipital-retromastoid craniectomy for excision tumour	
14	Right Frontal Lobe	Glioma-Astrocytome WHO Grade III	2.0x2.4x2.0	F	43/M	Redo Right Frontal Craniotomy for excision tumour	
15	Right Frontal Lobe	Glioma-Oligodendroglioma WHO Grade II	7.0x6.0x6.5	F	35/F	Right Fronto-temporal Craniotomy for Excision Tumour	
16	Left CP Angle	Vestibular Schwannoma	3.8x3.4x3.6	5, 3	54/M	Left Suboccipital Craniectomy for Excision Tumour	
17	Right Superior Cerebellar	Hemangioblastoma	2.0x2.5x2.2	1	42/M	Right SuboccipitalVHL Syndrome craniectomy for excision tumour	
18	Right Petroclival	Meningioma - WHO Grade III	5.2x6.2x4.6	5	58/M	Redo Right Temporal Craniotomy for Excision of Tumour	
19	Right CP Angle	Schwannoma (Lower CN)	1.3x0.8x1.0	3	54/F	Right Suboccipital Craniectomy for excision Tumour	With Lyme Disease/Chronic Depession
20	Left CP Angle	Vestibular Schwannoma	2.8x3.8x3.5	5, 3	58/M	Left Suboccipital Craniectomy for Excision Tumour	
21	Right Cerebellum	Glioblastoma - WHO Grade IV	4.5x3.3x3.0	5	59/M	Redo suboccipital craniectomy for excision tumour	
22	Right CP Angle-Jugulare Formamen	Paraganglioma Grade I	4.9x2.7x3.4	5, 1, Fellow	44/F	Redo Right Translabyrinthine Exposure for excision Tumour	
23	Right Fronto-Parietal	Meningioma - WHO Grade I	4.0x3.1x3.1	5, 3	34/F	Redo Posterior Paracentral Craniotomy for Excision Tumour	
24	Right CP Angle	Vestibular Schwannoma	2.0x2.0x1.9	5, Fellow	58/F	Right Translabyrinthine Exposure for Excision Tumour	
25	Tuberculum sellae-clival	Meningioma - WHO Grade I	3.6x2.4x3.4	5, 3	52/F	Redo Left Pterional Craniotomy for Excision Tumour	
26	Left Frontal	Meningioma - WHO Grade II	3.1x3.2x4.0	5, 1	84/M	Redo Left Frontal Craniotomyfor Excision Tumour	
27	Right Cerebellar	Hemangioblastoma	1.9x1.6x1.5	5, 1	44/M	Redo suboccipital craniectomy for excision tumour	VHL Syndrome
28	Left Frontal	Meningioma - WHO Grade II	1.3x2.5x1.3	3	65/F	Bifrontal Scalp Incision for Left Frontal Craniotomy for Excision Tumour	
29	Brainstem (Left Lateral Medulla)	Cavernous Angioma	1.6x1.4x2.1	5, Fellow	31/M	Suboccipital Craniectomy for Excision Brainstem Cavernous Angioma	
30	Left Parietal-Occipital/Parafalx	Meningioma - WHO Grade I	2.9x2.5x2.5	2	58/M	Left Occipital Craniectomy for Excision Tumour	RUMARY Colin
31	Right CP Angle	Vestibular Schwannoma	2.6x2.2x2.7	5	74/M	Right Suboccipital Craniectomy for excision Tumour	
32	Right Trigeminal Neuralgia	NA	NA	5	40/F	Right Suboccipital Craniectomy for Microvascular Decompression	
33	Right midbrain	Cavernous Angioma	1.8x1.5x1.8	5, Fellow, 1	58/F	Left Temporal Craniotomy for Excision Tumour-Cavernous Angioma	
34	Left CP Angle	Vestibular Schwannoma	1.5x1.0x0.8	Fellow, 5	64/M	Left Suboccipital Craniectomy for Excision Tumour	
35	Right Frontal Lobe	Meningioma - WHO Grade I	1.4x1.1x1.7	Fellow, 5	65/F	Right Frontal Craniotomy for excision tumour	
36	Right Frontal	Glioblastoma - WHO Grade IV	2.5x1.8x1.4	Fellow, 5, 1	67/M	Redo Right Frontotemporal Craniotomy for Excision Tumour	
37	Left CP Angle	Vestibular Schwannoma	5.5x3.0x3.5	6, 5, Fellow	31/F	Left Suboccipital Craniectomy for Excision Tumour	
38	Left Parietal/Parasaggital	Meningioma - WHO Grade II	3.4x2.4x2.2	6, 5	71/F	Redo left Paracentral Craniotomy for Excision Tumour	
39	Left CP Angle	Vestibular Schwannoma	2.8x2.0x2.2	6, 3	53/M	Redo Left Suboccipital Craniectomy for Excision tumour	
40	Right CP Angle	Vestibular Schwannoma	2.2x2.1x2.1	6, 2	66/F	Right Suboccipital Craniectomy for Excision Tumour	
41	Right Temporal Lobe	Glioma - Oligodendroglioma WHO Grade II	3.1x2.0x2.0	6, 5, Fellow	66/M	Redo Right Temporal Craniotomy for Excision of Tumour	
42	Right Hemifacial spasm	NA	NA	5, fellow	81/M	Redo Right Suboccipital Craniectomy for Microvascular Decompression-hemifacial spasm	Short SF
43	Cervical Spine	NA	NA	6	45/F	C4-C5 Anterior Cervical Discectomy & Fusion	
44	Right CP Angle	Vestibular Schwannoma	1.7x1.8x1.8	5, Fellow	61/F	Right Suboccipital Craniectomy for Excision Tumour	
45	Right Frontal-Parietal	Arterio-Venous Fistula	3.0x6.3x3.0	5, Medical Student	26/M	Right Paracentral Craniotomy for Excision Dural AV Fistula and Evacuation of Parietal lobe hematoma	Hematoma (Clot) size
46	Left Parietal	Meningioma - WHO Grade I	6.2x5.8x4.9	6, 3, Fellow	68/F	Left Parietal Craniotomy for Excision Tumour	
47	Right Temporal Lobe	Glioblastoma - WHO Grade IV	6.5x4.2x4.2	3, Fellow	67/M	Right Temporal Craniotomy for Excision Tumour	
48	Left CP Angle	Vestibular Schwannoma	0.9x0.5x0.4	6, 2, Fellow	64/M	Left Translab exposure for excision of tumour (CP Angle)	
49	Midline Posterior Fossa	Large Foramen Magnum-Arachnoid Cyst	6.8x3.2x6.1	6	31/F	Suboccipital Craniectomy and Duroplasty	
50	Left CP Angle	Vestibular Schwannoma	4.2x1.6x2.5	Fellow	69/M	Redo Left Suboccipital Craniectomy for Excision tumour - intracapsular removal	

The multimedia supplementary files for the software and device structure is available at https://github.com/smartforceps/supplementary/.

The reader can **show** any code chunk by clicking on the *code* button. We chose to make the default for the code hidden since we: (a) wanted to improve the readability of this document; and (b) assumed that the readers will not be interested in reading every code chunk.

The snippet below documents the list of **R** libraries that were used in this research. For convenience, we used the pacman package since it allows for installing/loading the needed libraries in one step.

Code

Data Recording

Data is generated through strain gauge sensors placed along the prongs of a bipolar forceps. The technology is designed and manufactured within OrbSurgical, ltd. in partnership with Bissinger, Germany. The calibrated data is analyzed and recorded in realtime through our software platform which is overviewed in the snippet below. Please navigate through different items in the tabs for various pages in the software.

SmartForceps Software

Home Page | Adding New Case | Download Forceps and Hospital Data Page | Data Monitoring Page | Data Upload Page

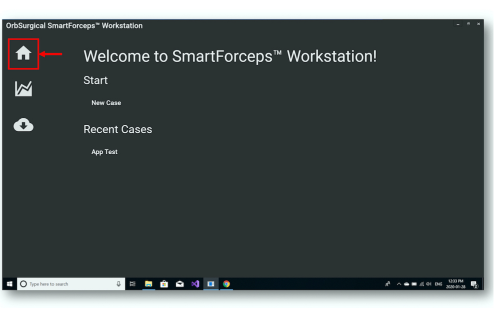

Software Demo

Below is a short demo of our software showing the force profiles in realtime with a high force error warning system (with 0.8 N threshold) and a voice recognition module to identify the keywords for different surgical tasks.

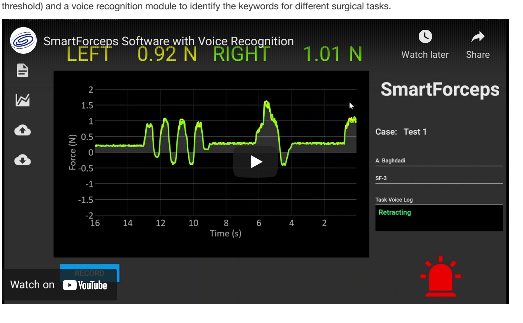

Orbsurgical SmartForceps Web Application

SmartForceps comes with a web application to manage the data, device characteristics (e.g. calibration factors), and medical center and surgeon information. This platform is designed in a way to manage the data to and from the cloud through integration into the software.

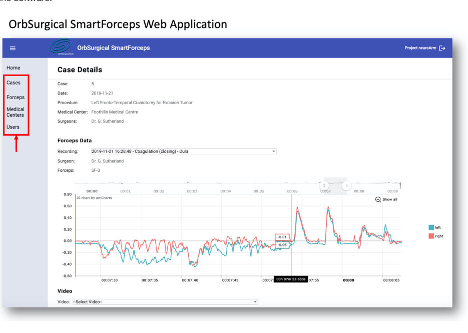

Data Processing

Data was extracted and analyzed using **R** programming platform. In the snippet below, we import the ".txt" files obtained from our Azure data cloud and the ".xlsx" file containing each task information (e.g. time stamp, task name, surgeon name, and remarks of potential intraoperative force errors) extracted from the surgical team voice recorded during each case. The imported data are structured as **R *dataframes***.

We have extracted various features from the segmented task force data in each prong. A combination of average, minimum, or maximum value of features for each prongs were included in the future analysis. These time-series features included:

1. **Force Duration**: duration of force application in one task segment.
2. **Force Average**: average of force values in one task segment.
3. **Force Max**: maximum of force values in one task segment.
4. **Force Min**: minimum of force values in one task segment.
5. **Force Range**: range of force values in one task segment.
6. **Force Median**: median of force values in one task segment.
7. **Force SD**: standard deviation of force values in one task segment.
8. **Force CV**: coefficient of variation of force values in one task segment.
9. **Force Mean CI (0.95)**: confidence interval on the mean with 95% probability.
10. **Force Data Skewness**: the extent to which the force data distribution deviates from a normal distribution.
11. **Force Data Skewness 2SE**: the significance of skewness in force data based on dividing by 2 standards errors (significant when > 1).
12. **Force Data Kurtosis**: the extent to which the force data distribution is tailed in a normal distribution.
13. **Force Data Kurtosis 2SE**: the significance of kurtosis in force data based on dividing by 2 standards errors (significant when > 1).
14. **Force Data Normality**: Shapiro-Wilk test of normality in force data distribution.
15. **Force Data Significance of Normality**: significance of Shapiro-Wilk test of normality.
16. **Force Peak Value**: peak force value in one task segment.
17. **Force Peak Counts**: number of force peaks in one task segment.
18. **1st Derivative SD**: standard deviation for the first derivative of the force signal in one task segment.
19. **Force Signal Flat Spots**: maximum run length for each section of force time-series when divided into ten equal-sized intervals.
20. **Force Signal Frequency**: dominant time-series harmonics extracted from Fast Fourier Transform (FFT) of force value in one task segment.
21. **Force Cycle Length**: average time length of force cycles in one task segment.
22. **Force Signal Trend**: force time-series trend in one task segment.
23. **Force Signal Fluctuations**: force time-series fluctuation index in one task segment.
24. **Force Signal Spikiness**: force time series spikiness index (variance of the leave-one-out variances of the remainder component) in one task segment.
25. **Force Signal Linearity**: force time-series linearity index (from Teräsvirta's nonlinearity test) in one task segment.
26. **Force Signal Stability**: force time-series stability index (variance of the means) in one task segment.
27. **Force Signal Lumpiness**: force time-series lumpiness index (variance of the variances) in one task segment.
28. **Force Signal Curvature**: force time-series curvature index in one task segment (calculated based on the coefficients of an orthogonal quadratic regression).
29. **Force Signal Mean Shift**: force time-series largest mean shift between two consecutive windows in one task segment.
30. **Force Signal Variance Shift**: force time-series largest variance shift between two consecutive windows in one task segment.
31. **Force Signal Divergence**: force time-series divergence index in one task segment (largest shift in Kulback-Leibler divergence between two consecutive windows).
32. **Force Signal Stationary Index**: force time-series stationary index around a deterministic trend in one task segment (based on Kwiatkowski-Phillips-Schmidt-Shin (KPSS) unit root test with linear trend and lag one).
33. **Force Signal Entropy**: force time-series forecastability in one task segment (low values indicate a high signal-to-noise ratio).
34. **First Autocorrelation Minimum**: time of first minimum of the autocorrelation function in force time-series signal from one task segment.
35. **First Autocorrelation Zero**: time of first zero crossing of the autocorrelation function in force time-series signal from one task segment.
36. **Autocorrelation Function E1**: first autocorrelation coefficient from force time-series signal in one task segment.
37. **Autocorrelation Function E10**: sum of the first ten squared autocorrelation coefficients from force time-series signal in one task segment.

Among these feature list, the best subset will be selected for the subsequent analysis based on statistical tests to monitor their representation power in different surgeon skill and task categories. The aim was to have the best explain of the patterns and behaviors for force profiles over the timespan of each data segment. To find accurate force peaks within each task segment, the signals were smoothed by passing through a digital 4st order Butterworth low-pass filter with a cutoff frequency of 0.1 Hz. Further, the outlier segmented data were identified based on 1st and 99th percentiles of either maximum force, minimum force or task completion time from all trials of the expert surgeon as <1% error was assumed to occur by experienced surgeons. The force segments for which the maximum force peak, minimum force valley, or task completion time exceeded the upper threshold (99th percentile) or fell short of the lower threshold (1st percentile) were labeled as outliers and removed (~11% were removed). The clean engineered features serve as a baseline for hand-crafted feature-based surgical skill classification.

The surgical tasks were classified as 5 main categories:

1. **Retracting**
2. **Manipulation**
3. **Dissecting**
4. **Pulling**
5. **Coagulation**

Code

Force Profiles

Below are the interactive figures of the force profiles for all 50 cases categorized in 5 common surgical tasks categories. The graph can highlight the differences in completion time and range of forces across the 5 surgical tasks.

SmartForceps Left and Right prong data output:

Left Prong Force | Right Prong Force

Code

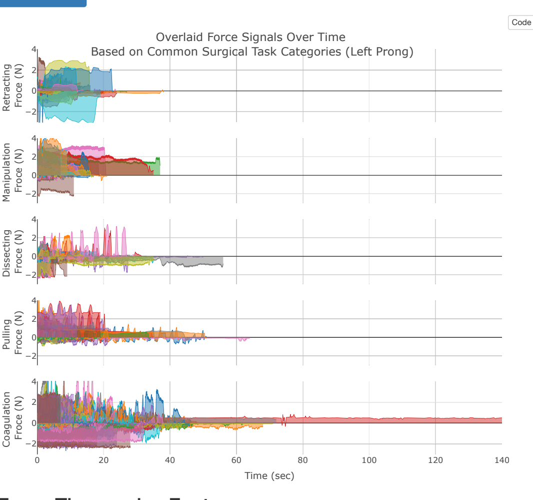

Force Time-series Features

Below are the interactive figures of the force time-series features extracted from all 50 cases categorized in 5 different tasks. The reader can see the relationship between different skill levels and across different tasks. The interactive figure can show detailed statistical results by mouse hover.

Multiple Time-series Features extracted: Please navigate through the dropdown List.

Force Duration

Code

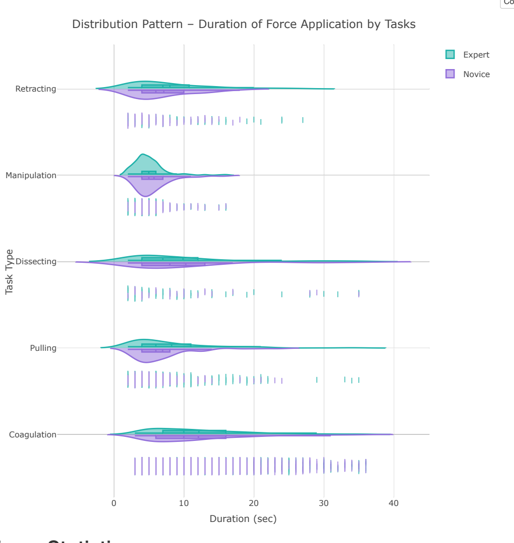

Force Statistics

In the snippet below, we provide the detailed statistics of force extracted features across various tasks for the surgeons.

Code
Code

Summary Statistics of Different Force Features

Table below is the summary of force statistics across different surgical tasks. Summary statistics were extracted for each task and surgeon experience that included the number of force segments and mean (SD) of the force features across all available segments.

Multiple Time-series Features extracted: Please navigate through the dropdown List.

Force Duration

Code

Statistics Summary of Duration.Force

Experience	Task	variable	n	mean	sd
Expert	Coagulation	Values	1108	12.149	7.169
Expert	Pulling	Values	192	8.250	6.315
Expert	Manipulation	Values	210	4.981	2.335
Expert	Dissecting	Values	64	9.875	8.514
Expert	Retracting	Values	71	8.000	5.931
Novice	Coagulation	Values	977	12.077	7.262
Novice	Pulling	Values	111	6.964	4.402
Novice	Manipulation	Values	86	5.733	2.707
Novice	Dissecting	Values	25	10.280	8.739
Novice	Retracting	Values	51	7.137	4.262

Test Results for the Effects of Task and Experience

ANOVA Test

To get the pattern of force data in various conditions under investigation, independent measures two-way ANOVA was performed that simultaneously evaluates the effect of experience and task type as two different grouping variables on the continuous variable of tool-tissue interaction force. Below is the table of ANOVA test results.

Code

Pairwise comparisons using ANOVA test

variable	Effect	DFn	DFd	F	p	p<.05	ges
Duration.Force	Experience	1	2885	0.321	5.71e-01		1.11e-04
Duration.Force	Task	4	2885	93.376	0.00e+00	•	1.15e-01
Duration.Force	Experience:Task	4	2885	0.913	4.55e-01		1.00e-03
Mean.Force	Experience	1	2885	97.230	0.00e+00	•	3.30e-02
Mean.Force	Task	4	2885	317.325	0.00e+00	•	3.06e-01
Mean.Force	Experience:Task	4	2885	10.810	0.00e+00	•	1.50e-02
Max.Force	Experience	1	2885	53.311	0.00e+00	•	1.80e-02
Max.Force	Task	4	2885	179.994	0.00e+00	•	2.00e-01
Max.Force	Experience:Task	4	2885	2.488	4.20e-02	•	3.00e-03
Min.Force	Experience	1	2885	2.100	1.47e-01		7.27e-04
Min.Force	Task	4	2885	64.270	0.00e+00	•	8.20e-02
Min.Force	Experience:Task	4	2885	3.874	4.00e-03	•	5.00e-03
Range.Force	Experience	1	2885	23.288	1.50e-06	•	8.00e-03
Range.Force	Task	4	2885	79.223	0.00e+00	•	9.90e-02
Range.Force	Experience:Task	4	2885	4.647	9.69e-04	•	6.00e-03
Median	Experience	1	2885	97.990	0.00e+00	•	3.30e-02
Median	Task	4	2885	399.432	0.00e+00	•	3.56e-01
Median	Experience:Task	4	2885	22.856	0.00e+00	•	3.10e-02
SD	Experience	1	2885	32.098	0.00e+00	•	1.10e-02
SD	Task	4	2885	184.986	0.00e+00	•	2.04e-01
SD	Experience:Task	4	2885	8.915	4.00e-07	•	1.20e-02
Coef.Variance	Experience	1	2885	0.206	6.50e-01		7.13e-05
Coef.Variance	Task	4	2885	0.788	5.33e-01		1.00e-03
Coef.Variance	Experience:Task	4	2885	0.082	9.88e-01		1.14e-04
CI.Mean.0.95	Experience	1	2885	30.771	0.00e+00	•	1.10e-02
CI.Mean.0.95	Task	4	2885	352.906	0.00e+00	•	3.29e-01
CI.Mean.0.95	Experience:Task	4	2885	20.070	0.00e+00	•	2.70e-02
Skewness	Experience	1	2885	0.050	8.22e-01		1.75e-05
Skewness	Task	4	2885	109.521	0.00e+00	•	1.32e-01
Skewness	Experience:Task	4	2885	11.668	0.00e+00	•	1.60e-02
Skewness.2SE	Experience	1	2885	1.816	1.78e-01		6.29e-04
Skewness.2SE	Task	4	2885	87.533	0.00e+00	•	1.08e-01
Skewness.2SE	Experience:Task	4	2885	6.197	5.80e-05	•	9.00e-03
Kurtosis	Experience	1	2885	20.327	6.80e-06	•	7.00e-03
Kurtosis	Task	4	2885	24.051	0.00e+00	•	3.20e-02
Kurtosis	Experience:Task	4	2885	2.840	2.30e-02	•	4.00e-03
Kurtosis.2SE	Experience	1	2885	17.653	2.73e-05	•	6.00e-03
Kurtosis.2SE	Task	4	2885	16.265	0.00e+00	•	2.20e-02
Kurtosis.2SE	Experience:Task	4	2885	2.483	4.20e-02	•	3.00e-03
Normtest.Wilk	Experience	1	2885	124.940	0.00e+00	•	4.20e-02
Normtest.Wilk	Task	4	2885	141.328	0.00e+00	•	1.64e-01
Normtest.Wilk	Experience:Task	4	2885	2.271	5.90e-02		3.00e-03
Normtest.Wilk.p	Experience	1	2885	12.382	4.40e-04	•	4.00e-03
Normtest.Wilk.p	Task	4	2885	2.072	8.20e-02		3.00e-03
Normtest.Wilk.p	Experience:Task	4	2885	0.492	7.42e-01		6.82e-04
Force.Peaks	Experience	1	2772	18.582	1.68e-05	•	7.00e-03
Force.Peaks	Task	4	2772	42.878	0.00e+00	•	5.80e-02
Force.Peaks	Experience:Task	4	2772	0.639	6.35e-01		9.21e-04
Peaks.Count	Experience	1	2885	1.897	1.69e-01		6.57e-04
Peaks.Count	Task	4	2885	129.419	0.00e+00	•	1.52e-01
Peaks.Count	Experience:Task	4	2885	0.561	6.91e-01		7.77e-04
Diff.Force.SD	Experience	1	2885	1.220	2.69e-01		4.23e-04

Diff.Force.SD	Task	4	2885	50.792	0.00e+00	•	6.60e-02
Diff.Force.SD	Experience:Task	4	2885	5.559	1.86e-04	•	8.00e-03
Flat.Spots	Experience	1	2885	25.525	5.00e-07	•	9.00e-03
Flat.Spots	Task	4	2885	93.222	0.00e+00	•	1.14e-01
Flat.Spots	Experience:Task	4	2885	3.571	7.00e-03	•	5.00e-03
Frequency	Experience	1	2885	11.556	6.84e-04	•	4.00e-03
Frequency	Task	4	2885	183.826	0.00e+00	•	2.03e-01
Frequency	Experience:Task	4	2885	3.015	1.70e-02	•	4.00e-03
Period.Length	Experience	1	2885	2.549	1.10e-01		8.83e-04
Period.Length	Task	4	2885	1.148	3.32e-01		2.00e-03
Period.Length	Experience:Task	4	2885	0.733	5.70e-01		1.00e-03
Trend	Experience	1	2885	7.697	6.00e-03	•	3.00e-03
Trend	Task	4	2885	202.540	0.00e+00	•	2.19e-01
Trend	Experience:Task	4	2885	3.476	8.00e-03	•	5.00e-03
Fluctuation	Experience	1	2885	5.346	2.10e-02	•	2.00e-03
Fluctuation	Task	4	2885	71.519	0.00e+00	•	9.00e-02
Fluctuation	Experience:Task	4	2885	1.142	3.35e-01		2.00e-03
Spike	Experience	1	2885	0.215	6.43e-01		7.45e-05
Spike	Task	4	2885	9.823	1.00e-07	•	1.30e-02
Spike	Experience:Task	4	2885	11.104	0.00e+00	•	1.50e-02
Linearity	Experience	1	2885	5.146	2.30e-02	•	2.00e-03
Linearity	Task	4	2885	13.916	0.00e+00	•	1.90e-02
Linearity	Experience:Task	4	2885	2.128	7.50e-02		3.00e-03
Stability	Experience	1	2885	14.224	1.66e-04	•	5.00e-03
Stability	Task	4	2885	138.941	0.00e+00	•	1.62e-01
Stability	Experience:Task	4	2885	3.515	7.00e-03	•	5.00e-03
Lumpiness	Experience	1	2885	20.948	4.90e-06	•	7.00e-03
Lumpiness	Task	4	2885	14.972	0.00e+00	•	2.00e-02
Lumpiness	Experience:Task	4	2885	0.822	5.11e-01		1.00e-03
Curvature	Experience	1	2885	22.564	2.10e-06	•	8.00e-03
Curvature	Task	4	2885	329.413	0.00e+00	•	3.14e-01
Curvature	Experience:Task	4	2885	5.419	2.40e-04	•	7.00e-03
Mean.Shift	Experience	1	2885	45.191	0.00e+00	•	1.50e-02
Mean.Shift	Task	4	2885	118.278	0.00e+00	•	1.41e-01
Mean.Shift	Experience:Task	4	2885	4.570	1.00e-03	•	6.00e-03
Var.Shift	Experience	1	2885	57.677	0.00e+00	•	2.00e-02
Var.Shift	Task	4	2885	64.889	0.00e+00	•	8.30e-02
Var.Shift	Experience:Task	4	2885	6.905	1.58e-05	•	9.00e-03
Divergence	Experience	1	2885	22.963	1.70e-06	•	8.00e-03
Divergence	Task	4	2885	13.381	0.00e+00	•	1.80e-02
Divergence	Experience:Task	4	2885	0.495	7.40e-01		6.85e-04
Stationary.Index	Experience	1	2885	2.210	1.37e-01		7.65e-04
Stationary.Index	Task	4	2885	5.518	2.01e-04	•	8.00e-03
Stationary.Index	Experience:Task	4	2885	0.807	5.21e-01		1.00e-03
Entropy	Experience	1	2885	11.055	8.95e-04	•	4.00e-03
Entropy	Task	4	2885	199.946	0.00e+00	•	2.17e-01
Entropy	Experience:Task	4	2885	6.101	6.93e-05	•	8.00e-03
First.Min.Autocorr	Experience	1	2885	27.309	2.00e-07	•	9.00e-03
First.Min.Autocorr	Task	4	2885	181.829	0.00e+00	•	2.01e-01
First.Min.Autocorr	Experience:Task	4	2885	7.072	1.16e-05	•	1.00e-02
First.Zero.Autocorr	Experience	1	2885	0.003	9.54e-01		1.10e-06
First.Zero.Autocorr	Task	4	2885	7.014	1.29e-05	•	1.00e-02
First.Zero.Autocorr	Experience:Task	4	2885	0.272	8.96e-01		3.77e-04
Autocorr.Function.E1	Experience	1	2885	45.404	0.00e+00	•	1.50e-02
Autocorr.Function.E1	Task	4	2885	80.628	0.00e+00	•	1.01e-01
Autocorr.Function.E1	Experience:Task	4	2885	16.536	0.00e+00	•	2.20e-02
Autocorr.Function.E10	Experience	1	2885	2.683	1.02e-01		9.29e-04
Autocorr.Function.E10	Task	4	2885	39.920	0.00e+00	•	5.20e-02
Autocorr.Function.E10	Experience:Task	4	2885	1.092	3.59e-01		2.00e-03

Code

ANOVA Post-hoc Analysis

In this section, we provide the post-hoc analysis results for cases where the ANOVA test was significant across various task categories.

Multiple Time-series Features extracted: Please navigate through the dropdown List.

Force Duration

Code

ANOVA post-hoc analysis of Duration.Force

Experience	.y.	group1	group2	df	statistic	p	p.adj	p.adj.signif
Expert	Values	Coagulation	Pulling	2885	7.4405025	0.0000000	0.0000000	****
Expert	Values	Coagulation	Manipulation	2885	14.2078099	0.0000000	0.0000000	****
Expert	Values	Coagulation	Dissecting	2885	2.6386399	0.0083687	0.0836868	ns
Expert	Values	Coagulation	Retracting	2885	5.0557589	0.0000005	0.0000046	****
Expert	Values	Pulling	Manipulation	2885	4.8840257	0.0000011	0.0000110	****
Expert	Values	Pulling	Dissecting	2885	-1.6795133	0.0931603	0.9316033	ns
Expert	Values	Pulling	Retracting	2885	0.2685044	0.7883303	1.0000000	ns
Expert	Values	Manipulation	Dissecting	2885	-5.1133086	0.0000003	0.0000034	****
Expert	Values	Manipulation	Retracting	2885	-3.2806892	0.0010479	0.0104786	•
Expert	Values	Dissecting	Retracting	2885	1.6227926	0.1047430	1.0000000	ns
Novice	Values	Coagulation	Pulling	2885	7.6148742	0.0000000	0.0000000	****
Novice	Values	Coagulation	Manipulation	2885	8.4142753	0.0000000	0.0000000	****
Novice	Values	Coagulation	Dissecting	2885	1.3233792	0.1858142	1.0000000	ns
Novice	Values	Coagulation	Retracting	2885	5.1301399	0.0000003	0.0000031	****
Novice	Values	Pulling	Manipulation	2885	1.2787577	0.2010852	1.0000000	ns
Novice	Values	Pulling	Dissecting	2885	-2.2345534	0.0255230	0.2552298	ns
Novice	Values	Pulling	Retracting	2885	-0.1528180	0.8785525	1.0000000	ns
Novice	Values	Manipulation	Dissecting	2885	-2.9856188	0.0028538	0.0285384	•
Novice	Values	Manipulation	Retracting	2885	-1.1856761	0.2358479	1.0000000	ns
Novice	Values	Dissecting	Retracting	2885	1.9202904	0.0549197	0.5491971	ns

SmartForceps Dashboard App

A custom-built Dash-Plotly architecture developed in Python environment to construct an interactive web application for visualization and interpretation of data. This platform was interfaced with a progressive web application (PWA) to make it installable on mobile devices.

GO TO THE INTERACTIVE DATA APP:

SmartForceps Dash App
